# Development and psychometric testing of the Chinese version of the Resilience Scale for Southeast Asian immigrant women who divorced in Taiwan

**DOI:** 10.1371/journal.pone.0211451

**Published:** 2019-02-04

**Authors:** Shu-Fen Kuo, Wen-Hsuan Hou, Chia-Chi Chang, Yuan-Mei Liao, Sue-Yueh Cheng, Yu-Hua Chou, Yueh-Chen Yeh, Yen-Kuang Lin, I-Hui Chen

**Affiliations:** 1 School of Nursing, College of Nursing, Taipei Medical University, Taipei City, Taiwan; 2 Master Program in Long-term Care, College of Nursing, Taipei Medical University, Taipei City, Taiwan; 3 School of Gerontology Health Management, College of Nursing, Taipei Medical University, Taipei City, Taiwan; 4 Attending Physical Medicine and Rehabilitation, Taipei Medical University Hospital, Taipei City, Taiwan; 5 College of Interdisciplinary Studies, Taipei Medical University, Taipei City, Taiwan; 6 Research Center of Active Aging, College of Nursing, Taipei Medical University, Taipei City, Taiwan; 7 Institute of Clinical Nursing, School of Nursing, National Yang-Ming University, Taipei City, Taiwan; 8 Department of Nursing, Cardinal Tien Junior College of Healthcare and Management, Taipei City, Taiwan; 9 Department of Nursing, National Taichung University of Science and Technology, Taichung, Taiwan; 10 Research Center of Biostatistics, Taipei Medical University, Taipei City, Taiwan; Unviersity of Sheffield, UNITED KINGDOM

## Abstract

**Background:**

Only a few studies exist on the resilience of divorced women. Furthermore, relevant instruments for assessing the resilience of divorced immigrant Southeast Asian women are rare. Accordingly, the aim of this study was to develop and examine a new Resilience Scale-Chinese version (RS-C) that is specific to divorced immigrant Southeast Asian women in Taiwan.

**Methods:**

The study was conducted in two phases. In phase 1, 20 items were used to evaluate face and content validities. In phase 2, a cross-sectional study was conducted. In total, 118 immigrant women participated in this study and were recruited from three nongovernmental organizations providing services for immigrants in Taipei City and Miaoli and Chiayi Counties. Psychometric properties of the instrument (i.e., internal consistency, test–retest reliability, item-to-total correlation, construct validity, and convergent validity) were examined. Significance was set at *p* < 0.05 for all statistical tests.

**Results:**

The final 16-item RS-C resulted in a three-factor model. The three factors, namely personal competence, family identity, and social connections, were an acceptable fit for the data and explained 54.60% of the variance. Cronbach’s α of the RS-C was 0.85, and those of its subscales ranged from 0.77 to 0.82. The correlation value of the test–retest reliability was 0.87. The RS-C was significantly associated with the General Self-Efficacy scale and the Chinese Health Questionnaire-12.

**Conclusion:**

The RS-C is a brief and specific self-report tool for evaluating the resilience of divorced immigrant Southeast Asian women and demonstrated adequate reliability and validity in this study. This RS-C instrument has potential applications in both clinical practice and research with strength-based resiliency interventions. However, additional research on the RS-C is required to further establish its reliability and validity.

## Introduction

Many Southeast Asian women have migrated to Taiwan over the past two decades. Many married Taiwanese men in their late 20’s during the period of economic expansion when the government’s policy toward Southeast Asia was being developed. Moreover, the number of newly divorced immigrant women in Taiwan has increased annually. Approximately one in three couples in transnational marriages experience a divorce [1]. The high rate of divorce in transnational marriages can be attributed to the following several reasons. First, immigrant women encounter an acculturation process and psychological distress [2]. Second, according to the results of a survey of 101,000 Southeast Asian immigrant women, 35.9% and 46.5% were introduced to their spouses through matchmaking agencies and through friends and relatives, respectively. In addition, the families of these women were economically less well off, and there was an age difference of more than 12 years between the Taiwanese husbands and Southeast Asian women [3]. Third, Taiwanese husbands might not trust their Southeast Asian wives resulting in them lacking social support from their homeland, friends, and relatives [4]. Fourth, the extensive process of adaptation requires intensive use of economic and social resources, including considerable psychological and physical effort, irrespective of one’s views of the immigration process.

Southeast Asian women who marry Taiwanese men can make adjustments in their marriages and new life despite encountering a diverse set of problems caused by difficulties and loneliness [5]. Nevertheless, their overall levels of psychosocial well-being deteriorate due to the effects of detrimental cultural, socioeconomic, and psychological factors, such as language barriers, unfamiliar customs and attitudes, economic hardship caused by an unstable source of income or a lack of job skills, and a relative lack of support from both the public and their community in Taiwan. Moreover, divorced immigrant women might leave Taiwan if they do not have the legal right to the custody of their child. Thus, divorced Southeast Asian immigrant women experience multiple stresses related to their immigrant and divorced status. Accordingly, understanding the process of adaptation in divorced women from intercultural marriages is crucial because their well-being can be adversely affected throughout this process [6].

Psychological resilience is considered a protective mechanism that can help ameliorate the effects of negative stressors [7]. Resilience is related to better health and wellness [8]. Thus, resilience is a vital concept for divorced immigrant women. However, the effects of divorce on settled immigrant women have not been adequately investigated. Most instruments for measuring resilience, including the Resilience Scale [9], the Connor–Davidson Resilience Scale [10], and the Resilience Scale for Adults [11], have been developed based on a sample of the general population or patients with psychiatric conditions [12, 13]. The Resilience Scale is the most widely used instrument for measuring resilience in immigrant women; this scale was originally developed for older women who adapted successfully following a major life event [14]. Some aspects of resilience and integration processes were found to be different between divorced Southeast Asian immigrant women and divorced women in other populations owing to their different cultural and gender values [15]. For example, divorced Southeast Asian immigrant women are required to learn skills to manage the loss of marriage (e.g., financial difficulties, loss of the spouse or parental role, and loss of support from the natal family) and their tarnished reputation [15–17]. Therefore, a multidimensional resilience scale specific to divorced Southeast Asian immigrant women is required to understand the unique aspects of their resilience. This study was conducted to fill this gap in the literature. In this study, a resilience scale specific to divorced Southeast Asian immigrant women was developed.

### Literature review

Resilience is defined as a dynamic process in which psychological, social, environmental, and biological factors interact to enable individuals at any stage of their life to develop, maintain, or regain their mental health despite being exposed to adversities [18]. Resilient individuals are described as those who not only survive but also thrive after periods of stress or adversity because of a supportive person, self-esteem, and a sense of self-determination [19]. Low psychological resilience has been associated with depression [9, 20, 21], suicidality [22], health problems [23], and high stress levels [10].

Divorced people are more likely to be adversely affected because of their stressful experiences. For many people, marital dissolution means renegotiating parenting relationships, changing friendships and social networks, moving out of the house, and addressing psychological challenges [24]. A divorce causes a decline in economic and social resources, which in turn results in a decline in a person’s health status [6]. However, most people cope well and are resilient after a divorce [25]. The basic capacity for resilience of divorced women is bolstered by personal competence, family support, and social support. Divorced women have to rebuild their old lives into new ones to meet their individual needs [26]. Self-efficacy appears to play a vital role in resilience and is a core concept of perceived control among divorced people. Self-efficacy is related to psychological adaptation after marital disruption [27].

Evidence showed that demographic characteristics correlate with resilience. Older adults are more resilient than younger adults, particularly in terms of emotional regulation and problem solving [28]. In addition, a person’s level of education is associated with his or her competence [23]. Moreover, a study reported that low resilience was related to being unemployed, having a depressed mood, and being perimenopausal. In one study, being employed was positively associated with higher levels of personal competence, social competence, and family coherence [29]. Furthermore, work experience was associated with family coherence [30].

### Framework

**Fig 1:**
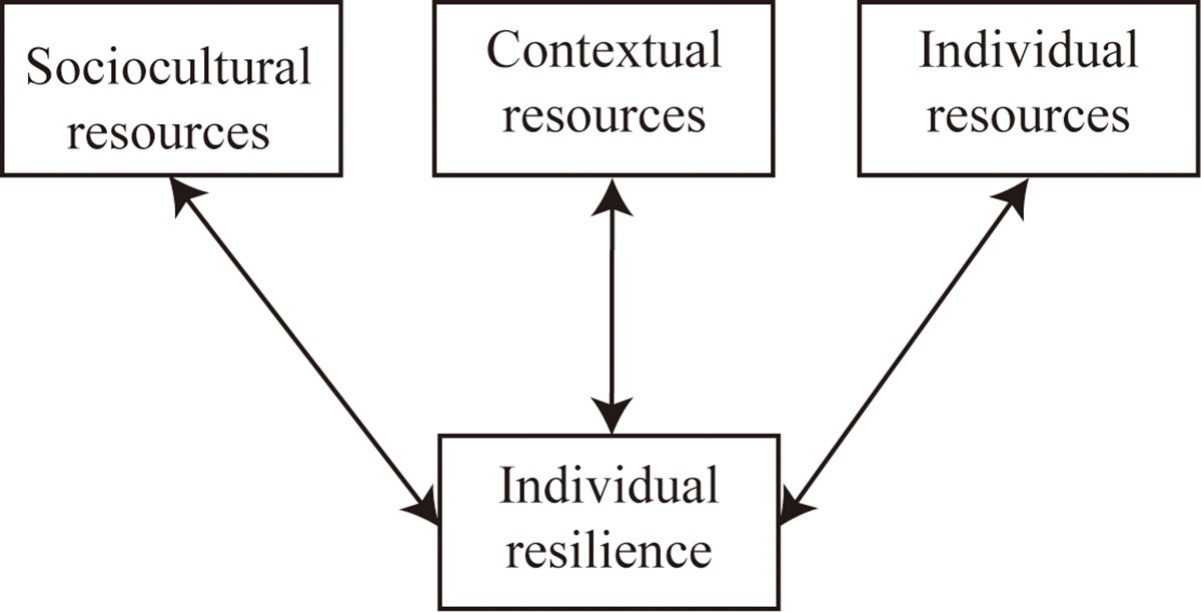
Framework of the Resilience Scale.

We constructed a theoretical model indicating the resilience process for divorced Southeast Asian immigrant women. In Fig 1, we have shown how a divorced Southeast Asian immigrant woman coping with stress may affect others in her immediate context as well as the broader social community; in turn, the immediate and broader sociocultural context may provide opportunities or create barriers to an individual’s positive coping strategies. With a high level of competence, an individual can meet a broad range of environmental demands that can result in positive effects.

This model consists of two main parts: individual resilience resources and individual interactions with an environment. Psychological resilience is conceptualized as a personality trait and is defined as the role of mental processes and behavior in promoting personal assets and protecting an individual from the potential negative effects of stressors. In addition, the resilience of Asian immigrant women is related to their social support and network and bicultural identity [31]. Resilience buffers the influence of detrimental cultural, socioeconomic, and psychological factors, such as language barriers, unfamiliar customs and attitudes, economic hardships caused by an unstable source of income or a lack of job skills, and the relative lack of support from both society and communities [32]. This concept emphasizes resilience at the individual level. However, divorce is a cascade of potentially stressful changes and disruptions in an adult’s social environment. Resilient individuals are more likely to maintain a positive attitude under stress, partly by accessing resources in their social environment.

We further applied an ecological system [33] to describe how an environment affects the resilience of divorced immigrant women. An ecological system determines the ways in which various systems interact with individuals to create individual outcomes [33]. Such systems include the microsystem, mesosystem, exosystem, and macrosystem. The microsystem focuses on direct interpersonal interactions between individuals and members of their immediate environment such as family. A divorced immigrant woman often encounters role strains and conflicts with family members (e.g., stabilizing income and rearing children). The mesosystem reflects interconnections and linkages between individuals and between individuals and systems, such as the original family, the parent-in-law’s family, friends, and peers. The exosystem includes organizations and social systems (e.g., legal, medical, or mental health). A divorced immigrant woman may have interactions in work places, neighborhoods, or social groups. In this developed model, the microsystem, mesosystem, and exosystem are combined as contextual resources because divorced Southeast Asian immigrant women may seek assistance from a friend (i.e., the microsystem) or from a nonprofit law organization in the host country, which can be conceptualized as a formal help resource (i.e., the exosystem); in the process of helping them, the friend or nonprofit law organization may help establish connections with other formal systems (such as the legal system), and the staff may work with them to help them access more informal support in their lives (i.e., the mesosystem). The macrosystem, referring to sociocultural resources, comprises societal norms, expectations, and beliefs that form the broader social environment [34].

## Methods and materials

### Two-phase design

This was an instrument validation study performed using a cross-sectional survey. To develop items for measuring resilience and assessing its psychometric properties, a two-phase design was used. In phase 1, we developed initial items and then evaluated their face and content validities. In phase 2, we examined the scale’s reliability and validity. Ethical approval was obtained from the research ethics review board of our university (TMU-JIRB no. 201302035) prior to recruitment. The purpose of the study was explained to the participants, and interviewers obtained informed consent from them. The participants were also informed that their responses were anonymous and that participation was voluntary.

#### Phase 1: Item development

**Item generation**

Items were generated based on the semi-structured interviews of Southeast Asian immigrant women. The main interview question, “What experiences did you have in Taiwan with family, friends, and work after you divorced?” was generated based on the theoretical framework. To increase the likelihood that the participants would have had sufficient experience with adapting to their new life after divorce, purposive sampling was employed. A small number of informants were recruited through an immigrant service center in New Taipei City. Women who (1) were aged ≥20 years, (2) had provided consent for inclusion in the study, (3) could communication in Mandarin or Taiwanese, (4) were divorced, and (5) were from Vietnam, Thailand, or Indonesia were included. Women were excluded from the study if (1) they had any history of mental illness such as depression or bipolar disorder or (2) they were protected by laws from domestic violence once more. Eleven women who had divorced their Taiwanese husbands participated in the study. The average age of the participants was 38.8 (range = 30–50) years, and they had fewer than three children on average. Their period of residency in Taiwan averaged 12.7 (range = 7–20) years at the time of the interview. Table 1 lists the demographic characteristics of the participants. Data were analyzed by performing a content analysis. The steps of the content analysis were as follows. First, transcribed interviews were read several times to identify and categorize meaningful units in all the interviews. Second, thematic codes across the interviews were confirmed. Third, another qualitative researcher from the nursing field was invited to independently categorize a random selection of manuscripts, and the reliability coefficient of kappa was 0.7 between two researchers. Finally, 56 descriptions and concepts were summarized into main findings by three independent raters (authors), and 20 items were produced (Table 2).

**Table 1:** Demographics of the sample in phase 1 (*N* = 11).

No.	Age (years)	No. of children	Country of origin	Educational level in country of origin	Residency in Taiwan (years)	Child custody
A	30	1	Thailand	Senior high school	9	no
B	34	0	Thailand	Senior high school	14	no
C	37	2	Vietnam	Junior high school	16	yes
D	42	2	Indonesia	Senior high school	20	yes
E	50	0	Thailand	Elementary school	10	no
F	50	0	Thailand	Elementary school	15	no
G	34	2	Thailand	Senior high school	7	no
H	36	2	Vietnam	Junior high school	9	yes
I	40	1	Indonesia	Elementary school	15	no
J	38	3	Vietnam	Elementary school	14	yes
K	36	2	Vietnam	Junior high school	11	yes

**Table 2:** The Resilience Scale-Chinese version.

Item
1. I have sufficient language capacity.
2. When I feel stressed, I can stabilize myself in my own way.
3. I believe that in the face of difficulties, everything will eventually pass smoothly.
4. If the result is not as good as expected, I tell myself, “well, it is OK, it will pass”.
5. I can learn according to my own needs.
6. I can pre-plan things and complete them in order.
7. At home, I can make my own decisions.
8. My ex-husband takes care of me and is concerned about me.
9. My family-in-law has a stable economic situation.
10. At home, the parents-in-law take care of me like a daughter.
11. I would be very sad if I separated from close family members.
12. For my child’s sake, I will try to stay here.
13. What I did for my family can be approved.
14. I try not to give my original family members reasons to worry about my family.
15. I would like to save money for my family.
16. I accommodate all of the family’s requests.
17. I have friends who will listen to and support me.
18. I receive help from the government or nongovernmental organizations, if necessary.
19. I can get substantial help from my friends.
20. I have good connections with close friends.

**Face and content validities**

The face validity of the newly developed 20-item resilience scale-Chinese version (RS-C) was determined by enrolling five additional Southeast Asian immigrant women. The participants indicated if the items were relevant and if they felt comfortable completing the questionnaire. Content validity was examined by 10 experts from government organizations, nongovernmental organizations (NGOs), and nursing, sociology and psychology departments (S1 Table). These experts reviewed each item for its completeness, clarity, and consistency based on the questionnaire’s focus. All experts were asked to rate the relevance of each item on a 4-point scale (1 = not relevant to 4 = very relevant). Using this content validity index (CVI) based on a 4-point scale, we calculated the validity of the content of each item. A CVI score ranging from 0.7 to 1 represented a relevant item [35]. On the basis of the experts’ recommendations and discussions among the members of the research team, eight items with scores ranging from 0.7 to 0.9 were reworded. The remaining items with a score of 1 were not changed.

### Phase 2: Psychometrics

Psychometric properties comprise internal consistency, test–retest reliability, item-to-total correlation, construct validity, and convergent validity. Overall, this study comprised a convenience sample of 118 participants recruited from NGOs that provide services for immigrants in Taipei City and Miaoli and Chiayi Counties. The inclusion and exclusion criteria were the same as those used in phase 1. Construct validity was evaluated using exploratory factor analysis (EFA). The sample size was considered adequate as per the minimum requirement of five participants per variable [36]. In addition to assessing the demographic characteristics, convergent validity was tested by examining self-efficacy, which is positively related to resilience [37]. Another aspect of convergent validity was examined by comparing the scores of the women with and without psychological morbidity. Psychological morbidity was chosen to assess convergent validity because a study reported that women with psychological morbidity were less resilient than those without it [20].

#### Measures

**The RS-C version**

The final version of the RS-C includes 20 items. Approximately 10 minutes is required to complete this questionnaire. Responses are rated on a 5-point Likert scale ranging from 1 “strongly disagree” to 5 “strongly agree.” The total score ranges from 20 to 100, with a higher score indicating greater psychological resilience.

**General Self-Efficacy Scale**

The Chinese version of the General Self-Efficacy Scale (CSE) based on Bandura’s self-efficacy theory was originally developed by Zhang and Schwarzer to assess self-efficacy in response to stressful events [38]. The CSE contains 10 questions, and self-reported answers are rated on a 4-point scale (1 = not at all true to 4 = exactly true). The total score ranges from 10 to 40, with a higher score indicating greater self-efficacy. The scale has been widely used in 27 countries, including China, India, Japan, and South Korea. The CSE had satisfactory reliability and validity [38]. Cronbach’s α coefficient for this study was 0.86.

**Chinese Health Questionnaire-12**

The Chinese Health Questionnaire-12 (CHQ-12) was selected to measure the immigrant women’s levels of anxiety and depression to evaluate the concurrent criterion-related validity of the RS-C. The CHQ-12, originally developed by Cheng and Williams [39], is based on social and cultural characteristics of Chinese and their ways of expressing psychological difficulties, including anxiety, somatic symptoms, depression, and poor family relationships. The CHQ-12 is a short, self-administered screening instrument used to identify minor psychiatric morbidity in community and primary care settings. Items are scored on a 4-point Likert scale. The total score ranges from 0 to 12, and a cutoff value of ≥3 indicates minor psychiatric morbidity. The sensitivity and specificity of this tool in predicting cases of psychiatric morbidity were 69.6% and 98.4%, respectively [40], and Cronbach’s α was 0.84 [41]. Cronbach’s α in this study was 0.87.

**Data analysis**

Internal consistency was assessed using Cronbach’s α coefficient. Cronbach’s α of ≥0.7 for newly developed scales and item-to-total correlations of up to 0.3 is recommended [35]. All items were evaluated using the critical ratio (CR) and discrimination index (DI). A CR of >5 and a DI of >0.5 are recommended [42]. The EFA was performed using a principal axis factoring method, followed by varimax rotation, to estimate the number of factors. The Kaiser–Meyer–Olkin (KMO) measure of sampling adequacy was ≥0.74, and Bartlett’s test of sphericity was significant (*p* < 0.05), indicating that the items were adequate for factor analysis. Kaiser’s eigenvalue of >1, a scree plot, and the conceptual meaning of items were used to determine the number of components to be retained [36]. After EFA testing, the Velicer’s minimum average partial (MAP) test and a parallel analysis were conducted to confirm the number of factors. The number of factors to be extracted was determined by the step number that resulted in the smallest average squared partial correlation. The parallel analysis involved the comparison of actual eigenvalues with random data eigenvalues. The number of eigenvalues obtained from actual data should be greater than the 95th percentile (and mean) of random data eigenvalues [43–46]. An independent two-sample *t* test, Pearson’s correlation test, and analysis of variance were performed to assess convergent validity. Scheff’s post-hoc comparison test was used. Significance was set at *p* < 0.05 for all statistical tests. All statistical analyses were conducted using SPSS (version 18.0) for Windows (SPSS, Chicago, IL, USA).

## Results

### Sociodemographic characteristics of the sample

The sample comprised 118 women, of whom 33.05%, 42.37%, and 24.58% were from Thailand, Vietnam, and Indonesia, respectively. The age of the participants ranged from 27 to 66 (mean = 40.33 and standard deviation [SD] = 8.40) years, and the mean age of the Thai, Vietnamese, and Indonesian women was 45.51 (SD = 7.92 and range = 32–66), 36.86 (SD = 5.01 and range = 28–53), and 39.37 (SD = 10.29 and range = 27–65) years, respectively. Their mean length of stay in Taiwan was 13.59 (SD = 6.23) years, and their length of stay ranged from 4 to 44 years at the time of the interview. Approximately 80% of the women had received less than or equal to junior high school education in their country of origin, and 40.68% of the women had had not received schooling in Taiwan. The participants’ demographic characteristics are listed in Table 3.

**Table 3:** Sociodemographic characteristics of participants in phase 2 (*N* = 118).

Demographic variable	*n* / Mean (SD)	%	Range
Nationality			
Thailand		33.05	
Vietnam		42.37	
Indonesia		24.58	
Age (years)	40.33 (8.40)		27~66
Years in Taiwan	13.59 (6.23)		4~44
Education received in country of origin			
None	9	7.63	
Elementary school	51	43.22	
Junior high school	34	28.81	
High school	19	16.10	
≥University	5	4.24	
Education received in Taiwan			
None	48	40.68	
Elementary school	51	43.22	
Junior high school	8	6.78	
High school	5	4.24	
≥University	6	5.08	
Job			
Full-time	58	49.15	
Part-time	52	44.07	
Unemployed	8	6.80	
Ex-husband’s job			
Full-time	57	48.31	
Part-time	33	28.00	
Unemployed	28	23.73	
Monthly family income^a^			
<NT$20,000	39	33.05	
NT$20,001~30,000	75	63.56	
NT$30,001~40,000	4	3.39	
Resilience scale Chinese version	54.70 (8.49)		34~73
Self-efficacy	25.57 (5.78)		12~39
Psychological morbidity			
Healthy	63	53.40	
Stressed	55	46.60	

SD = standard deviation.

^a^The average exchange rate in July 2015 was US$1.00≈New Taiwan (NT)$30.795.

### CR

The following four items had a CR of <5 and a DI of <0.5 (Table 4): Item 1 (CR = 2.480; DI = 0.41), Item 7 (CR = 4.173; DI = 0.30), Item 11 (CR = 3.186; DI = 0.26), and Item 18 (CR = 4.048; DI = 0.30). Hence, these four items were deleted. Finally, a 16-item RS-C was retained.

**Table 4:** Characteristics, DIs and CRs for the items in the 20-item RS-C.

Item	Mean (SD)	Kurtosis	Skewness	High Resilience Group		Low Resilience Group		DI	CR
				Mean(SD)	PL	Mean(SD)	PL		
1	3.46(0.97)	-0.97	-0.11	3.91(0.90)	0.79	3.31(1.06)	0.38	0.41	2.48
2	3.81(0.91)	0.79	-0.93	4.44(0.66)	0.91	3.16(0.95)	0.38	0.57	6.33
3	3.76(0.84)	0.69	-0.83	4.29(0.62)	0.91	3.21(0.89)	0.40	0.51	5.03
4	3.78(0.89)	0.85	-0.89	4.32(0.72)	0.91	3.09(0.95)	0.40	0.51	5.76
5	3.63(0.88)	-0.65	-0.13	4.38(0.69)	0.88	2.84(0.62)	0.12	0.76	9.40
6	3.55(0.96)	-0.63	-0.18	4.47(0.61)	0.94	2.78(0.70)	0.12	0.82	10.38
7	3.25(1.15)	-0.80	-0.39	3.76(0.98)	0.64	2.59(1.26)	0.34	0.30	4.17
8	2.64(1.03)	-0.19	0.30	3.29(1.00)	0.76	2.00(0.71)	0.25	0.51	6.06
9	2.76(1.01)	-0.76	0.24	3.35(0.84)	0.82	2.41(1.04)	0.31	0.51	5.06
10	2.68(1.00)	0.01	0.53	3.44(0.99)	0.82	2.13(0.55)	0.21	0.61	6.71
11	3.88(0.88)	1.12	-0.92	4.29(0.71)	0.91	3.66(0.90)	0.65	0.26	3.19
12	3.82(1.04)	-0.16	-0.62	4.29(0.90)	0.85	3.13(0.97)	0.34	0.53	5.05
13	3.53(1.02)	0.14	-0.59	4.18(0.71)	0.82	2.63(1.08)	0.15	0.67	7.24
14	3.90(1.02)	0.92	-0.99	4.59(0.60)	0.94	3.19(0.96)	0.40	0.54	7.10
15	3.42(0.96)	-0.54	-0.32	4.12(0.68)	0.88	3.03(0.74)	0.21	0.67	6.19
16	3.30(1.02)	-0.98	0.07	4.00(0.92)	0.76	2.53(0.80)	0.15	0.61	6.89
17	3.47(0.77)	-0.39	-0.37	3.82(0.62)	0.70	2.81(0.69)	0.15	0.55	6.23
18	3.61(0.88)	-0.56	-0.31	3.91(0.66)	0.73	3.06(0.98)	0.43	0.30	4.09
19	3.33(0.87)	-0.99	-0.30	3.88(0.64)	0.97	2.56(0.66)	0.09	0.88	8.19
20	3.35(0.91)	-0.46	-0.33	3.94(0.54)	0.82	2.63(0.90)	0.15	0.67	7.09

DI = discrimination index; CR = critical ratio; SD = standard deviation; PL = popular level.

### Validity testing

#### Factor analysis

**Fig 2:**
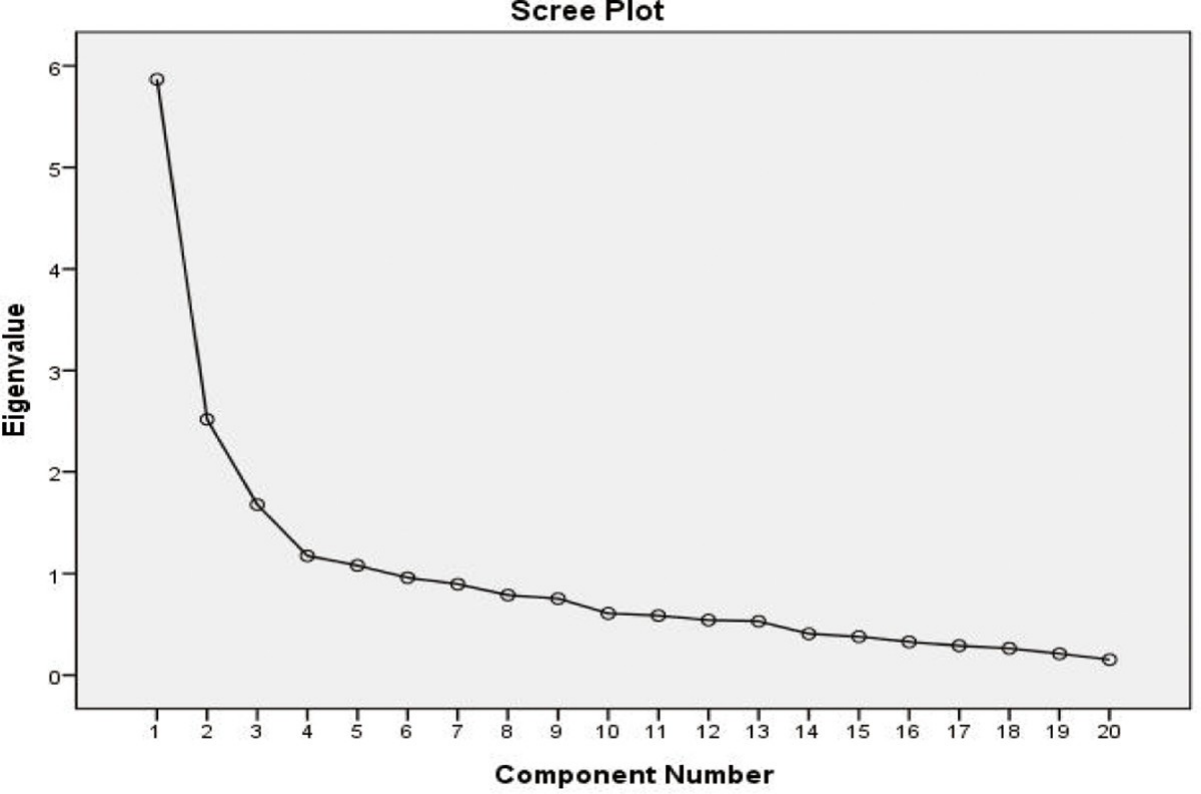
Scree plot.

The KMO coefficient was 0.78, and the Bartlett’s test of sphericity value was 710.706 (*p* < 0.001), indicating that the properties of the correlation matrix justified performing a factor analysis. According to the visual interpretation of a scree plot (Fig 2) and Kaiser’s criterion for eigenvalues of >1.4, three latent factors were extracted that explained 54.60% of the total variance. Table 5 illustrates the loading coefficients.

**Table 5:** Results of the exploratory factor analysis of the Resilience Scale-Chinese version (*N* = 118).

Item	Factor 1	Factor 2	Factor 3
**Personal competence**			
2.When I feel stressed, I can stabilize myself in my own way.	0.64		
3. I believe that in the face of difficulties, everything will eventually pass smoothly.	0.56		
4. If the result is not as good as expected, I tell myself, “well, it is OK, it will pass”.	0.64		
5. I can learn according to my own needs.	0.71		
6. I can pre-plan things and complete them in order.	0.75		
12. For my child’s sake, I will try to stay here.	0.53		
13. What I do for my family can be approved.	0.54		
14. I try not to give my original family members reasons to worry about my family.	0.53		
15. I would like to save money for my family.	0.46		
16. I accommodate all of the family’s requests.	0.61		
**Family identity**			
8. My ex-husband takes care of me and is concerned about me.		0.72	
9. My family-in-law has a stable economic situation.		0.64	
10. At home, the parents-in-law take care of me like a daughter.		0.65	
**Social connections**			
17. I have friends who listen to and support me.			0.51
19. I can get substantial help from my friends.			0.42
20. I have good connections with close friends.			0.49

The first factor “personal competence” accounted for 32.61% of the total variance with 10 items (with an eigenvalue of 5.21). The factor “personal competence” was related to displaying an individual’s abilities (e.g., doing something under stress and maintaining family commitments) and to trusting her own beliefs (e.g., looking beyond and letting sufferings go), indicating that individual resilience interacted with the individual and sociocultural resources of the theoretical framework. The second factor “family identity” explained 13.11% of the total variance with three items (with an eigenvalue of 2.09). The factor “family identity” was related to fostering family interactivity, such as care based on trust and respect under traditional Chinese values, which corresponded to the contextual resources of the theoretical framework. The third factor “social connections” explained 8.86% of the total variance with three items (with an eigenvalue of 1.41). The factor “social connections” was related to making new friends as psychological or substantial resource supports, which corresponded to the contextual resources of the theoretical framework. By using the MAP algorithm, we obtained the smallest average squared partial correlation and the smallest average 4^th^-power partial correlation of 0.0305 and 0.0025, respectively. A three-component model was suggested by MAP results because the variance in the correlation matrix represented systematic variance. The output of the parallel analysis showed that the first three eigenvalues obtained from actual data were the same as the corresponding first three median random data eigenvalues. The four eigenvalues obtained from actual data were lower than the fourth median random data eigenvalues. Therefore, the results of the MAP test and parallel analysis both suggested that a three-factor model should be retained for interpretation and subsequent rotation.

#### Criterion-related validity

The bivariate associations of sample characteristics with the RS-C total scale and the three subscales are shown in Table 6. The self-efficacy scale was positively associated with the RS-C (*r* = 0.51, *p* < 0.001), personal competence subscale (*r* = 0.47, *p* < 0.001), family identity subscale (*r* = 0.35, *p* < 0.001), and social connections subscale (*r* = 0.26, *p* = 0.003). Compared with the women with no psychological distress, the women with stress and high scores on the CHQ-12 demonstrated low scores on the RS-C total scale (with stress = 52.65 ± 8.96 and healthy = 56.49 ± 7.69; *p* = 0.014), personal competence subscale (with stress = 34.58 ± 6.58 and healthy = 38.12 ± 4.89; *p* = 0.001), and social connections subscale (with stress = 9.72 ± 2.02 and healthy = 10.52 ± 2.15; *p* = 0.042). No significant difference was noted in the family identity subscale (with stress = 8.34 ± 2.48 and healthy = 7.84 ± 2.60; *p* = 0.286). Accordingly, the criterion-related validity was proven.

**Table 6:** Bivariate associations between sample characteristics with the Chinese version of the Resilience Scale (RS-C) total scale and three subscales (*N* = 118).

Variable	Personal competence		Family identity		Social connections		RS-C	
	*F/t/r*	*p*	*F/t/r*	*p*	*F/t/r*	*p*	*F/t/r*	*p*
Nationality	2.98	0.054	1.97	0.144	6.98	0.001^c^	1.61	0.203
Years in Taiwan ^a^	0.18	0.045	-0.03	0.745	0.12	0.188	0.17	0.060
Education received in country of origin	5.36	0.001	3.55	0.009^d^	2.24	0.068	5.32	0.001^e^
Education received in Taiwan	2.55	0.043	2.84	0.027	0.72	0.576	1.71	0.152
Job	1.63	0.199	6.01	0.003^f^	3.09	0.049	3.51	0.033
Self-efficacy^a^	0.47	<0.001	0.35	<0.001	0.26	0.003	0.51	<0.001
Psychological morbidity^b^	-3.28	0.001	1.07	0.286	-2.06	0.042	-2.50	0.014

*F* = ANOVA, *t* = *t*-test, *r* = correlation.

^a^ A correlation was applied.

^b^ The *t*-test was applied.

^c^ Scheffe's test: Thailand vs. Vietnam *t* = 1.34 (*p* = 0.009); Thailand vs. Indonesia *t* = 1.64(*p* = 0.005).

^d^ Scheffe's test: Junior high school vs. Elementary school *t* = 1.76 (*p* = 0.037).

^e^ Scheffe's test: Junior high school vs. Illiterate *t* = 9.59 (*p* = 0.040); Junior high school vs. Elementary school *t* = 5.66 (*p* = 0.040).

^f^ Scheffe's test: Full-time vs. Unemployed *t* = 3.17 (*p* = 0.004); Part-time vs. Unemployed *t* = 2.58 (*p* = 0.024).

### Reliability testing

#### Internal consistency

Cronbach’s α coefficient of the RS-C was 0.85 for the overall scale, indicating moderate to high internal reliability. Cronbach’s α coefficients of the personal competence, family identity, and social connections subscales were 0.82, 0.79, and 0.77, respectively. All item-to-total correlations of the 16-item RS-C were >0.43 (range = 0.43–0.73) and were statistically significant (*p* < 0.001).

#### Test–retest reliability

Test–retest reliability was assessed in the 30 participants over a 2-week period, and the intraclass correlation coefficient of the RS-C was 0.87 (*p* < 0.01).

## Discussion

In this study, the RS-C was developed on the basis of our resilience framework and differed from the current resilience measures because it was specifically designed for identifying the resilience process in divorced Southeast Asian immigrant women. The RS-C provides a more holistic view of resilience in divorced Southeast Asian immigrant women. A resilience framework with the ecological system for post-divorce families was established in Halabuza’s resilience model [34]. The difference between Halabuza’s resilience model and our model is that the experience of the stress process in Halabuza’s resilience model was based on the problems of multifamily structures, whereas the experience of the stress process in our resilience model was based on individual interactions with environments.

The three components of the RS-C were personal competence, family identity, and social connections. The factor “personal competence” showed that individual resilience interacted with contextual and sociocultural resources. These immigrant women maintained their Buddhist beliefs in a stressful environment and demonstrated these Buddhist beliefs in real life. In particular, the positive beliefs of individual resilience interacting with sociocultural resources buffer the stress value of being “well behaved” and are significantly related to overcoming barriers [32, 47]. The factor “family identity” showed that the participants’ caring relationships improved based on trust and acceptance in the immigrant ex-family. The factor “social connections” related to receiving additional support from close friends [16, 48]. The RS-C focuses on the unique aspect of the interaction of individual resilience with contextual resources that are close to life environments among divorced Southeast Asian immigrant women [16, 17].

This study provides data on psychometric support for the RS-C scale. We found acceptable internal consistency and reliability for the RS-C overall and its three subscales. The construct validity of the RS-C was also strongly supported by the theoretical model, factor analysis, and correlation analysis. Two items were excluded from the personal competence subscale. One was “I have sufficient language capacity”. Of the immigrant women enrolled in our study, 40.68% had received no schooling in Taiwan. Multiple languages are spoken in Taiwanese families; therefore, the experience of language capacity might not be a clear concept for this population. The other was “I would be very sad if I separated from close family members.” The participants considered their children the most important family members. After the divorce, the women still had opportunities to contact their children if they had custody or received help from their ex-husbands. Hence, this might not be a clear concept for this population. One item was removed from the family identity subscale (i.e., “At home, I can make my own decisions”). In East Asian cultures, the daughter-in-law is not the main decision maker if parents-in-law live with the family. Communication is often unidirectional in an ethnic Chinese family; thus, this might not be a clear concept for this population. One item was excluded from the social connections subscale (i.e., “I receive help from the government or nongovernmental organizations, if necessary”). A study found that only few women in intercultural marriages received help from the government or NGOs [48]; therefore, this might not be a clear concept for this population.

Our results consistently and strongly supported relationships among the RS-C, self- efficacy, and CHQ-12. The divorced women with psychological morbidity scored lower on the RS-C than did the divorced women without psychological morbidity. In particular, the women with psychological morbidity and those without it scored significantly differently in personal competence and supportive resources. The participants whose resilience was lower and who were exposed to stressors (e.g., a divorce or loss of family-in-law relationships) had lower competence in coping with adversity [48]. Moreover, a study showed that depressed women of ethnic minorities were less likely to perceive a need for mental health care in the new country [49]. Therefore, to foster those immigrants’ resilience, reduce their stress levels, and improve their mental health, it is imperative for the host country to provide supportive resources and opportunities for them to develop their individual competence in adaptive coping techniques.

This newly developed instrument can help health care providers identify immigrant women who have lower resilience and require greater health support. Several studies have shown that divorced immigrants have high needs for personal, family, and community assistance [48, 50]. Health care providers can routinely include the assessment of immigrants’ resilience levels in their practice settings, such as in health centers or home visits. Moreover, health care providers can play a vital role in educating and communicating with women in intercultural marriages; thus, immigrant women can be empowered with appropriate medical care services and mental support.

The present study has several limitations. First, we could not conduct confirmatory factor analysis for examining the construct validity of the RS-C because of a small sample size. Additional research on the RS-C is required to further establish its validity. Second, the study relied on self-reported data collected at one time point; thus, follow-up data would be of value in examining whether resilience results are consistent in demonstrating dispositional traits of individuals and can help health care professionals understand changes in resilience in Southeast Asian immigrant women over time. Third, our sample is not representative of all women who experienced divorce in intercultural marriages in Taiwan. It would be useful to determine whether the original nationality, education level, and employment exert different effects on resilience, because these results can be first-hand information for service providers, including immigration officers, social workers, physicians, and nurses. Accordingly, counseling services can provide early assistance and promote the quality of the marriage. Additional studies enrolling a larger representative sample would be beneficial.

## Conclusions

This study demonstrated that the RS-C is a feasible, reliable, and valid measurement tool for evaluating resilience in women who experienced divorce in intercultural marriages in Taiwan. This scale is brief and can be easily used to measure the resilience of immigrants in an Asian context. This multidimensional tool can provide essential information in clinical settings. In particular, women who experience divorce in intercultural marriages in Taiwan have multiple tasks, such as adapting to new roles and work, and require more social services [48, 50, 51]. Although not all health care providers have a direct experience with divorce, they can develop skills and sensitivity for mental health care based on empirical research on resilience. In Taiwan, there has been an increase in immigrant women from other parts of Asia over the last two decades; however, the literature specific to resilience is scant. By gathering baseline information using the RS-C, health care professionals can have a better understanding of mental health care and develop psychological interventions to foster resilience among immigrant women.

## Supporting information

S1 Table Experts characteristics. (DOC)
