# Supplementary material for: Development and psychometric testing of the Chinese version of the Resilience Scale for Southeast Asian immigrant women who divorced in Taiwan
Source: PLoS One. 2019 Feb 4;14(2):e0211451. doi: 10.1371/journal.pone.0211451 (PMC6361505; doi:10.1371/journal.pone.0211451)
Supplement: S1 Table — (DOC) [file pone.0211451.s001.doc]

**Supplementary Table 1. Experts c**haracteristics

| Department | Title | Professional field | Age |
| --- | --- | --- | --- |
| Government organizations | | | |
| Immigration Department North District　Affairs Brigade | Deputy captain | Immigrant & social | 38 |
| Department of Civil Affairs Taipei City Government | Chief | Social & police | 40 |
| Non-governmental organizations | | | |
| Taiwan Immigrant Family Service Association | Chairman | Immigrant & police | 50 |
| Wanhua Hospital Social Work Room | Director | Social & police | 50 |
| International Social Service- Taiwan Branch | Social worker | Social worker | 36 |
| Banqiao Haishan Public Care Center in New Taipei City | Social worker | Social worker | 45 |
| Nursing department | | | |
| National Cheng Kung University | Professor | Nursing | 50 |
| Tzu Chi University | Assistant professor | Nursing | 52 |
| Sociology department | | | |
| National Taipei University | Professor | Sociology | 62 |
| Psychology department | |  |  |
| Taipei Medical University | Associate professor | Psychology | 52 |
